# A tale of 10 European centres – 2010 APOSSM travelling fellowship review in ACL surgery

**DOI:** 10.1186/1758-2555-4-27

**Published:** 2012-07-28

**Authors:** Yee Han Dave Lee, Ryosuke Kuroda, Jinzhong Zhao, Kai Ming Chan

**Affiliations:** 1 Department of Orthopedic Surgery, Changi General Hospital, 2 Simei St 3, Singapore, 529889, Singapore; 2 Department of Orthopedic Suregry, Kobe University Graduate School of Medicine, 7-5-2 Kusunoki-cho, Chuou-Ku, Kobe, 650-0017, Japan; 3 Department of Arthroscopy Surgery, Shanghai No 6 Peoples Hospital, Shanghai, 200233, China; 4 Department of Orthopedics & Traumatology, The Chinese University of Hong Kong, Prince of Wales Hospital, Hong Kong SAR, PR China

## Abstract

The purpose of ESSKA- APOSSM Travelling fellowship is to better understand the epidemiology, management and surgical techniques for sports across continents. There has been a progressive evolution in ACL reconstruction and there is variation in technique in ACL reconstruction amongst the most experienced surgeons in different continents. During this one month fellowship, we saw various ACL reconstruction techniques using different graft sources, with a variety of graft fixation methods, with the common aim of recreating an anatomical ACL reconstruction.

## Introduction

The Anterior Cruciate Ligament(ACL) is well studied ligament and there has been a progressive evolution in ACL reconstruction technique that is documented in the literature. The industry has aided this evolution with the improvements in implant design and biomaterial sciences. There is variation in technique in ACL reconstruction amongst the most experienced surgeons in different continents.

The purpose of ESSKA- APOSSM Travelling fellowship was to gain a better understanding into sports surgery- its epidemiology, management and surgical techniques across continents. As participants of this month long travelling fellowship, we had the opportunity to visit ten top European Sports centres in seven countries.

## Methods

During this travelling fellowship, we had seen numerous ACL reconstructions – each different in surgical technique. Each of the centre that we visited were recoginsed as a premier European sports knee reconstruction and the surgeons there performed an average of 150 to 200 ACL reconstructions annually. It represents a good dichotomy of how ACL reconstruction is performed in the European continent and we felt it was beneficial to review this as it crystalises where we are with ACL reconstruction in 2011.

## Results

 1. All the grafts in the ACL reconstructions we saw were autograft tissue. We had 6 cases where Hamstring autograft tendon and 4 cases where bone patella tendon bone (BTB) graft was used. Table 1 summarises the breakdown of graft types during this fellowship.

 2. The majority of centres perform a single femur and tibial tunnel ACL reconstruction, with the aim of recreating the ACL anatomical footprint. There was one case of double bundle ACL reconstruction that was performed in one of the centres.

 3. We saw that no notchplasties were performed in ACL footprint preparation, except in BTB ACL reconstruction and chronic ACL reconstructions.

 4. All the femoral tunnels were drilled independently through the anteromedial portal except for two cases; a BTB ACL reconstruction with transtibial femoral tunnel drilling and an outside–in femoral tunnel drilling for revision ACL reconstruction.

 5. The majority of hamstring graft fixations on the femoral side was accomplished with cortical suspensory fixation. One hamstring graft ACL reconstruction was performed as an all-inside technique with bioabsorbable interference screw fixation on the femur. For hamstring grafts, the tibial fixation were all achieved with bioabsorbable interference screws. In two centres, we note the practice of backup the tibia fixation with staples in addition to the tibia screws.

 6. For BTB ACL reconstructions, fixation on the femur and tibia were achieved with metal or bioabsorbable interference screws, except for one centre; where they used a direct cortical button technique. All the BTB ACL reconstructions were fixed on the tibia side with interference screws, either metal or bioabsorbable. Table 2 sumarises the femoral and tibia fixation methods used.

 7. The majority of ACL reconstruction were performed surgery at approximately one month after the injury. However, we are aware that some centres in the alpine region treating ski injuries perform acute ACL reconstruction within a week of injuries.

 8. We saw was the importance all European centres placed on preserving the meniscus in ACL reconstruction surgery. Meniscus that were reparable were repaired with all-inside meniscal repair methods, inside –out and outside-in methods.

 9. There were 2 combined cases of ACL reconstruction and high tibial osteotomy for chronic ACL injuries with varus alignment. Both procedures started with the osteotomy and was followed by drilling of the tunnels for ACL reconstruction. We did not note any compromise to the positions of the tunnels during the ACL reconstruction as a results of the combined surgery.

 10. There were 3 Revision ACL Reconstructions performed for traumatic reinjuries. All the cases were single stage autograft ACL revisions; 2 cases used ipsilateral BTB autograft and the other used contralateral Hamstring autograft as all 3 patients had previous hamstring ACL reconstruction.

**Table 1:** Breakdown of graft sources

	**Primary ACL Surgery (Cases)**	**Revision ACL Surgery (Cases)**
Hamstring autograft	5	1
Bone Patella Tendon Bone autograft	2	2

**Table 2:** Types of graft fixation methods in the femur

	**Endobutton**	**Bioabsorbable Screws**	**Metal Screws**	**Remarks**
Hamstring	4	1	0	1 Evolgate Swing bridge
BTB	1 (direct)	1	2	

## Discussion

### Graft Choice

In European centres, the use of allograft tissue is not prevalent. Allograft tissue is not readily available in many countries and only permitted for use when all other autograft sources are exhausted. From our literature review summarised in Table 3, two graft choice trends in ACL surgery are apparent. Over the last 5 to ten years, we see the increased use of allograft tissue in ACL surgery in American centres. In addition, the American surgeons are generally biased towards the use of Bone Patellar Tendon Bone autografts as compared to Hamstring autograft, compared to European surgeons [1-6].

**Table 3:** Graft Choices among surgeons worldwide from 2000-2010

**Literature**	**Autograft Hamstring**	**Autograft BTB**	**Allograft**	**Remarks**
Mirza F et al. [7]	32%	59%		Remaining used other ACL reconstruction techniques
2000 Survey of Canadian Surgeons				
Feller et al. [4]	50%	50%		
2001 Survey Australian Surgeons				
Kapoor et al. [5]	37%	63%		
2002 Survey of 192 UK Knee surgeons				
Campbelll J et al. [2]	25%	70%	5%	Mainly US Surgeons
2004 ACL Study Group				
Dequin et al. [3]	32%	46%	22%	
2006 AOSSM Survey				
Barker JU [1]	11.1%	45.7%	43.1%	
2002-2006 HSS data				
Magnussen et al. [6]	44%	42%	13%	Remaining 0.6% used other autograft sources
2010 MOON Cohort US Surgeons				
Magnussen et al. [6]	60%	37%	0.1%	Remaining 3% used other autograft sources
2010 NKLR Norwegian Surgeons				

In addition, we note many European surgeons are comfortable with the use of both Bone patella tendon bone and Hamstring autografts for ACL reconstruction Over the last few years, European and Asia-Pacific centres have seen increased rates of Hamstring grafts as the primary graft source. This trend was seen during this travelling fellowship.

There are basic science and clinical studies that highlight the concerns of allograft tissue use in ACL reconstruction. Scheffler et al. found in a sheep model that there was delayed allograft incorporation at 6 and 12 weeks when compared to autograft. This differences was less distinct at 52 weeks. The authors felt that full weightbearing should be delayed in allograft ACL reconstruction [8]. Singhal et al. reports of high failure rates of 38% after ACL tibialis anterior allograft surgery in their clinical study and cautioned the use of interference screw fixation and an accelerated rehabilitation protocol with allograft ACL reconstructions [9]. However, there are many other authors with 5 and 10 year clinical follow-ups of allograft ACL reconstruction that have comparable clinical outcomes when compared to autograft reconstructions [10-12].

Biomechanical studies have shown that the various allograft and autograft sources used today surpass the native ACL, when compared for ultimate load to failure and stiffness [13-18]. Table 4 summaries this information. The BTB graft has the advantage of direct bone-to-bone contact for rapid incorporation. The BTB graft achieves direct attachment with Sharpey-like fibres found at the interface with the bone tunnel, resembling normal ACL at 12 weeks in a canine model [19]. Grana et al. found indirect healing between host bone and soft tissue when soft tissue hamstring grafts are used [20]. Rodeo et al. reported that Sharpeys fibres were identified by 12 weeks for soft tissue grafts, but they only reached maturity at 26 weeks [19]. Rehabilitation protocols have been designed to take this slower graft incorporation with soft tissue grafts into account.

**Table 4:** Biomechanical studies of the various graft choices

	**Ultimate Load (N)**	**Stiffness(N/mm)**	**Remarks**
Native ACL	2160	242	Woo et al. [13]
Doubled Hamstring Autograft	4140	807	Hamner et al. [14]
BTB autograft	2977	450	Cooper et al. [15]
BTB allograft fresh frozen	2252	633	Fiedeler et al. [16]
Soft tissue allograft fresh frozen	4122	625	Haut-Donahue et al. [17]
			Pearsall aw et al. [18]

Various authors have found that hamstring autografts have less anterior knee pain, extension deficits and progression to osteoarthritis, compared to BTB ACL reconstructions [21-23]. Wagner at al in their prospective matched analysis of hamstring autograft versus BTB ACL reconstruction found hamstring grafts superior in knee function and function, based on outcome scorings and instrumented laxity measurements [24]. However, Biau et al. in their metaanalysis of pooled data from 6 Randomised Controlled Trials (RCTs); concluded that there was less knee instability after BTB autograft ACL reconstruction than with hamstring autograft [25]. Other authors have found that there no difference between patients reconstructed with hamstring or BTB autografts at 5–10 year follow-up, based on knee function scores, instrumented laxity testing and repeat radiographs to look for progression of osteoarthritis [26-28]. Samuelsson K et al. in their systematic review of ACL surgery with special reference to graft type and surgical technique found that in terms of laxity and clinical outcomes, there was no differences between the bone patellar tendon bone and hamstring autografts [29].

### Double bundle ACL Techniques

Muneta et al. first described the technique of double bundle ACL reconstruction in 1999 [30]. There has been a trend towards double bundle ACL reconstruction among sports surgeons [31,32]. In cadaveric studies, it is shown that anatomic double bundle ACL reconstruction better restores the normal patellofemoral contact pressures [33], tibiofemoral joint pressures at low flexion angles [34], rotational stability [35], the anterior-posterior and medial-lateral laxities than single bundle reconstruction [36]. With the use of a robot sensor test system in cadaver knees, it has been also shown double bundle ACL reconstruction better restores the intact knee kinematics [37]. The differential anteromedial and posterolateral bundle tensioning in double bundle ACL reconstructions more closely replicate native ACL strain patterns [38].

Fu FH et al. reported good 2-year outcomes for 100 consecutive double bundle ACL reconstructions, based on knee function scores and instrumented laxity testing [39]. Other studies that compare double bundle ACL reconstructions against single bundle ACL reconstructions have not shown improved knee outcomes [40-42]. Similarly, Song EK et al. found that double-bundle ACL reconstructions had better intraoperative stability than single bundle ACL reconstructions; but both were similar in 2-year post-operative clinical outcomes and stabilities [43]. A Metaanalysis by Meredick comparing single-bundle versus double-bundle ACL reconstruction RCTs concluded no significant differences in instrumented laxity or pivot- shift testing [44].

With these non-conclusive data, some authors have suggested that the current knee outcome measures are not sensitive or precise enough [45]. The pivot shift test performed is different in the hands of different individuals. There has been a call for the use of more sensitive instruments advanced imaging modalities to detect early cartilage changes to compare outcomes.

### Anatomic ACL

**Figure 1:**
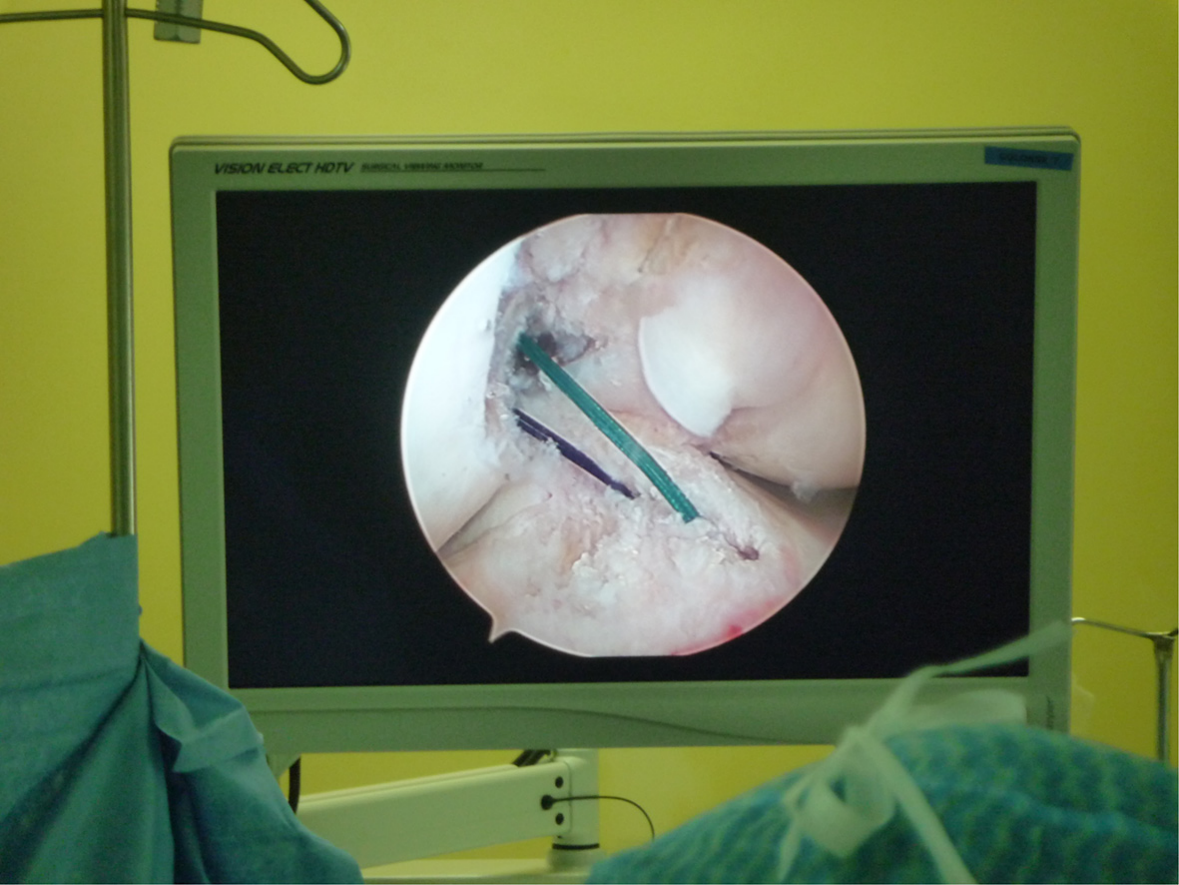
Anatomical ACL construction viewing from the anterolateral portal prior to grafts passed.

Anatomic ACL is defined as the functional restoration of the ACL to its native dimensions, collagen orientation and insertions sites [46,47]. This means using landmarks on the femur and tibia to re-establish the ACL footprint as accurately as possible. All the European centres that we visited practise the concept of an anatomical ACL reconstruction (Figure 1).

The research in the 1990s described the isometric position for the femoral attachment of the ACL graft and sports surgeons strived to achieve an isometric ACL reconstruction, often combined with a notchplasty [48,49]. Cadaveric and biomechanical studies have shown that the native ACL is non-isometric. It has been shown that a femoral tunnel position within the anatomical footprint of the ACL results in more normal knee kinematics than a tunnel position located for best graft isometry [50]. An anatomical femoral tunnel position is important to restore the normal kinematics of the knee as demonstrated by Abebe et al [51]. It is now believed that recreating the insertional footprint anatomically as well as the tension of the two bundle concept of the anterior cruciate ligament re-establishes the function of the anterior cruciate ligament.

Cadaveric studies have been performed to define the anatomical footprint of the ACL [52-55]. For the femoral footprint of the ACL, the lateral intercondylar ridge also known as Residents ridge is located on the medial wall of the lateral femoral condyle. Shino K et al. has demonstrated this landmark - the Resident’s ridge is readily identifiable on arthroscopy [56]. It runs from anterior to posterior with the knee in 90 degrees and seen from the medial portal. The lateral bifurcate ridge runs perpendicular to the lateral intercondylar ridge and is located between the AM and PL bundles. Many surgeons now identify the anatomy of the native femoral insertion during reconstruction and often no longer depend on guides as a reference.

One technical pearl that we have learnt from our visit to one of the centres was a reproducible method to locate the ACL femoral insertion site. They suggest that centre of the ACL insertion lies at a point 50% along a line drawn from the proximal articular cartilage border and the distal articular cartilage parallel to the tibial surface, with the knee at 90 degrees [57].

The tibial insertion of the ACL is taken as from the remnant of the ACL stump or referenced against the anterior insertion of the lateral meniscus. This is aligned with the anteromedial aspect of the ACL insertion. The posterolateral bundle of the ACL is aligned to the posterior attachment of the lateral meniscus [58]. Some surgeons reference the ACL insertion to a distance of approximately 7 mm anterior to the anterior edge of the PCL [59,60]. The criticism is that this may place the ACL tibial insertion too posterior. The transverse ligament coincides with the anterior edge of ACL tibial foot print, which considered as a new landmark for tibial tunnel positioning during anatomic ACL reconstruction [61].

In the ACL reconstruction, augumentation without resection of the ACL remnant using an autograft tendon is important new technique. The ACL remnant has the proprioceptive, biomechanical functions, and vascularity. More rapid vascularization from the ACL remnant to the grafted tendon and improved recovery of proprioceptive function with this technique can be expected. The utilities of this procedure are improved joint stability, position sense, and superior reported knee outcome scores [62,63].

### Notchplasty

Notchplasty had been previously advocated to reduce the amount of notch impingement and ACL graft injury. Some authors believe that this makes the reconstruction easier by visualising the posterolateral margin of the intercondylar space more clearly [64]. Van Eck et al. in their evidence based review of recent articles published on the anatomic ACL reconstruction, reported that only 12% of studies performed a notchplasty [65]. In the age of anatomic ACL reconstruction, performing a notchplasty seems to be less common and this was what we saw during this travelling fellowship.

There are three concerns in the literature with performing a notchplasty. LaPrade et al. found that in a canine model study that aggressive notchplasty caused cartilage changes seen on histology at six months, consistent with early arthritis [66]. Markolf et al. in their cadaveric study found that a notchplasty created unfavourable graft forces, graft elongation and failure. They recommended removing as little bone as possible during the notchplasty [67]. The other concern is the removal of osseous landmarks of the ACL femoral insertion and compromise anatomic placement [65].

### Femoral drilling

The literature now shows that transtibial femoral tunnel drilling has many drawbacks. Despite an ideal tibial tunnel position, Bedi et al. found in a cadaveric study that the femoral tunnel drilled transtibial was anterior and superior to the femoral footprint [68]. They concluded that independent anteromedial portal drilling allows accurate positioning in the centre of the native footprint. Alentorn-Geli et al. reported that anteromedial portal drilling for BTB ACL reconstructions significantly improved the anterior-posterior and rotational knee stability, functional outcome scores compared to the transtibial technique [69].

**Figure 2:**
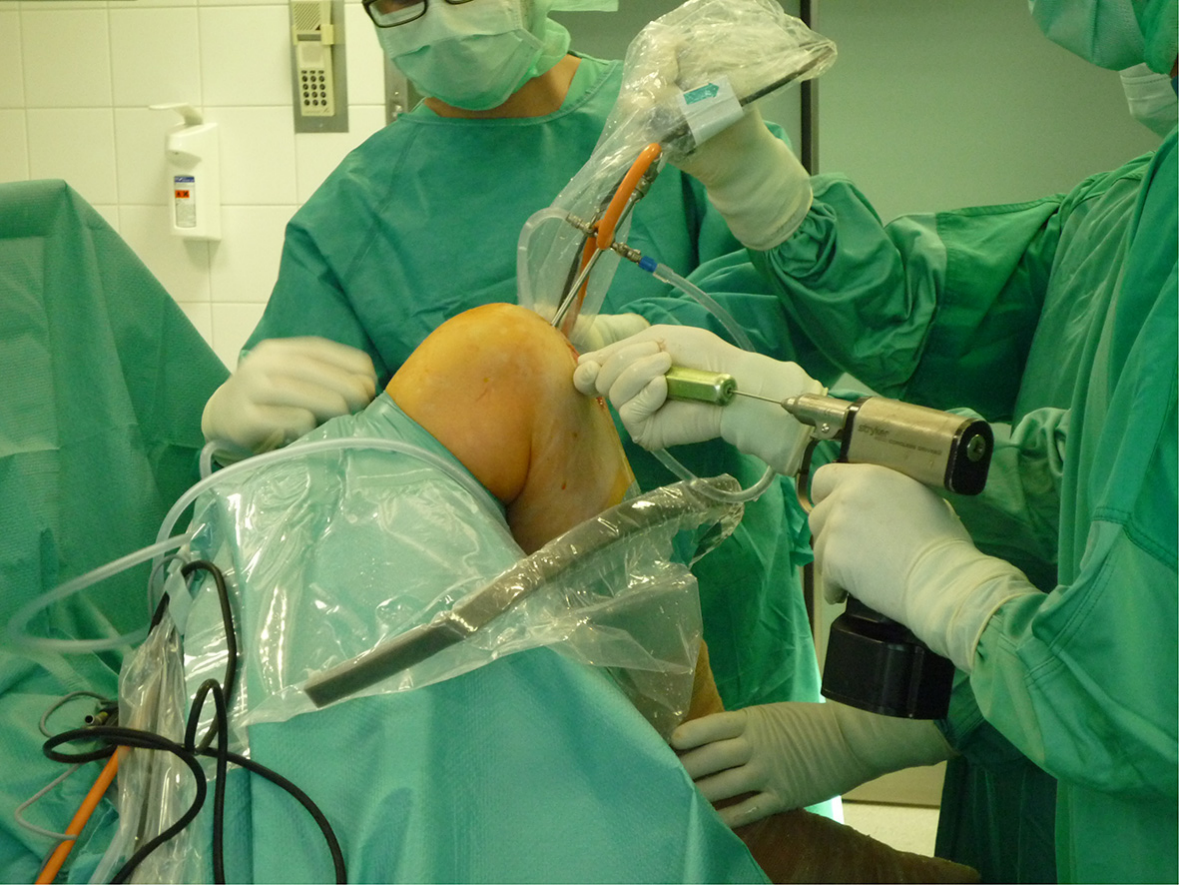
Drilling the femur tunnel through an anteromedial portal with the knee hyperflexed.

An audience poll at 2010 Fall AANA revealed that more than 50% of surgeons in the audience drilled their femoral tunnels through a transtibial technique. During this travelling fellowship, the majority of the femoral tunnels were drilled independently from the anteromedial portal (Figure 2). The key concerns with anteromedial portal drilling are: short femoral tunnel length, injury to the cartilage, injury to posterolateral knee structures and posterior wall blow-out [70-72].

One key technical pearl that we have learnt is the importance of hyperflexion with anteromedial portal drilling as alluded to by Nakamura et al [73]. All the surgeons that we visited drilled the femoral tunnel through the anteromedial portal did so with flexion beyond 120 degrees. It has been shown that anteromedial drilling has to be performed with hyperflexion of the knee [74]. Basdekis et al. has found that increasing the knee flexion when drilling from an anteromedial portal prevents femoral posterior wall blowout and gives longer femoral tunnel lengths [70].

Outside in femoral drilling was practised by surgeons for a long time. With surgeons revisiting independent drilling of femur and tibial tunnels in ACL reconstruction, many surgeons now use outside in drilling for femoral tunnels [75]. The benefits of outside-in drilling are: safety to the lateral knee structures, less risk of posterior wall blowout and longer femoral tunnel for graft fixation [75,76].

### Graft Fixation

The preference in The European centres we visited for femoral fixation of soft tissue hamstring grafts was suspensory cortical button fixation. Ahmad CS et al. found in a porcine biomechanical study that suspensory cortical femoral fixation had a higher ultimate failure load of 864 N and less graft slippage as compared to interference screw with a failure load of 539 N [77]. Various other authors have also shown that extracortical fixation has a mean load to failure of 700 N to 1150 N during biomechanical testing [78,79]. The failure load for the interference screw is close to 450 N, which is what Noyes terms the physiological load that the knee has to withstand [80]. The drawbacks of suspensory cortical fixation are: a less stiff fixation construct and the bungee effect of suspensory button fixation on graft tunnel widening [81-83]. However, Ma et al. found that at 2 years, aperture fixation with bioabsorbable interference screw did not lead to significant differences in clinical outcomes or reduced tunnel widening when compared to the cortical suspensory fixation [84].

On the tibial side, we saw the preference for the use of bioabsorbable interference screws for tibial fixation. Some authors report that metal screws had a higher rate of graft laceration compared to bioabsorbable screws in hamstring ACL reconstruction. [85,86] Moisala AS et el found that using certain bioabsorbable screw tibia fixation for ACL reconstruction led to 23% graft failure rates, compared to 6% with metal screws [87]. The meta-analysis that compared ACL reconstruction outcomes using bioabsobable and metallic screws, found no significant differences in functional outcomes or stability [88].

In two centres with hamstring ACL reconstruction, in addition to the use tibia bioabsorbable interference screws, we note the use of backup fixation with soft tissue staples. Walsh et al. in their porcine model biomechanical study found that soft tissue grafts fixed with a retroscrew backed-up with a suture button had higher ultimate failure loads and had stiffer constructs that grafts fixed with either the retroscrew or suture button alone [89]. Hill et al. in their randomised controlled study found that supplementary tibial staple fixation in female patients undergoing hamstring autograft ACL reconstruction with tibia interference screw fixation can reduce the knee laxity at 2-years awhen compared to tibia interference screw fixation alone [90]. These studies suggest benefit of the use of backup fixation in the tibia with selected patients.

### Tensiometer

**Figure 3:**
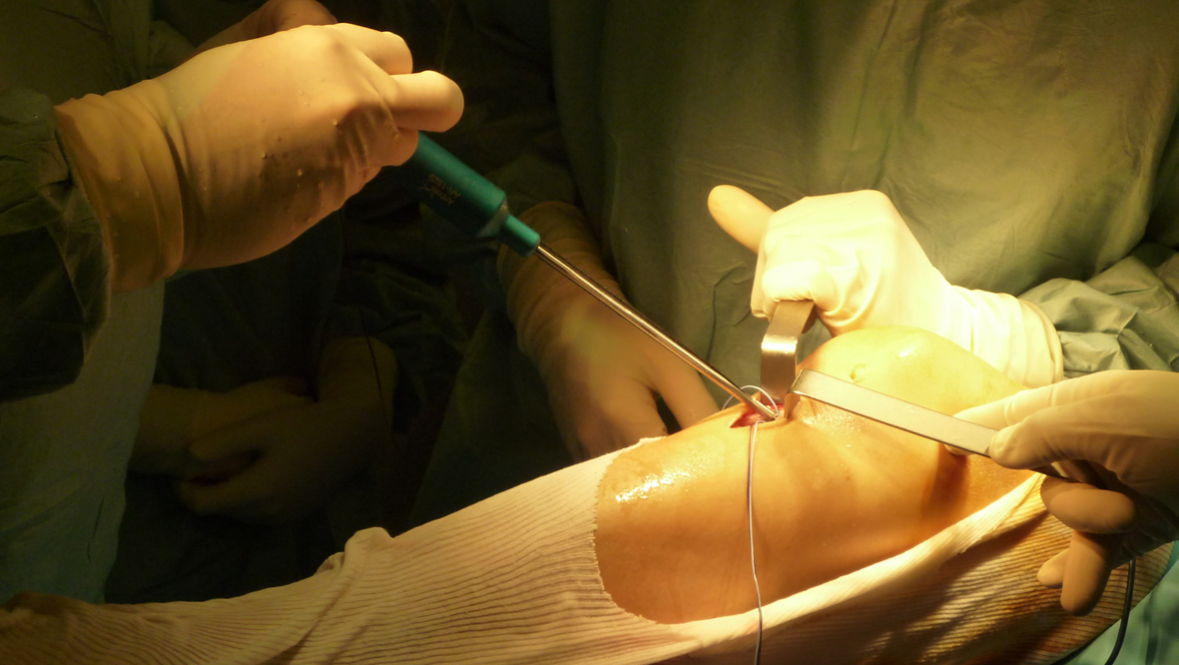
Using a tensiometer during final tensioning of the ACL with insertion of tibia interference screw.

High graft tension induces poor vascularity, myxoid degeneration and deterioration in graft mechanical properties in a canine model [91,92]. Conversely, low graft tension produces knee laxity, changes in knee kinematics and progressive deterioration of tendon mechanical properties [93,94]. Chang et al. has shown that using a tensioner during graft fixation provided superior soft tissue graft tunnel fixation when compared to manual tensioning [95]. During our travelling fellowship, we note the widepread use of tensioners for graft fixation during ACL reconstruction (Figure 3). This is an important technical pearl as the use of a tensiometer, as compared to manual tensioning is a critical step to ensure the longevity of a reconstructed ACL graft.

The optimal tension load and angle of flexion during tensioning has yet to agreed in the body of literature [96]. Numazaki et al. suggested that an initial tension of 20 N for patellar tendon grafts and going beyond 80 N for hamstring grafts is unnecessary [97]. Arnold MP et al. found that tensioning the graft at 10 N at 10 degree of flexion allows the graft to be taut in all positions of flexion while avoiding excessive tension [98]. Tatsuo et al. found that the ACL graft fixed at 20 degrees of flexion is most optimal [99].

### Meniscus in ACL

The preference of the European surgeons we had visited,for meniscal injuries during ACL reconstruction was meniscus repair. Spand et al. has shown in their cadaveric biomechanical study that the meniscus is a secondary stabiliser of the ACL and a menisectomy produces an increased strain on the ACL [100]. Thus, preserving the meniscus in ACL surgery helps to protect graft from subsequent failure. The literature has shown that meniscus deficient knees are exposed to increased articular contact pressures and typically progress to joint degeneration [101-103].

Magnussen et al. in their metaananalysis on the effect of the meniscus on ACL outcomes at more than 2 years follow-up found that all patients who underwent partial menisectomy developed radiographic changes [104]. In their 24-year follow up of ACL reconstructed patients, Pernin J highlights the importance of meniscus preservation to prevent osteoarthritis. They found that only 38% patients with an intact medial meniscus had osteoarthritis, compared to 68% of patients with a previous menisectomy who developed osteoarthritis [105]. Brophy et al. found that contrary to belief, an isolated ACL does not significantly shorten the career nor reduce the playing time of an National Football League (NFL) athlete. However, they found that an isolated menisectomy significantly reduces career length and playing time of an NFL player [106].

### Chronic ACL

Gerrit et al. have shown in their cadaveric knee study showed that an unstable ACL deficient knee with a varus thrust leads to an increased risk of ACL failure [107]. This emphasises the importance of simultaneous alignment correction in young patients with both chronic anterior knee instability and varus deformity - a combined high tibial osteotomy and ACL reconstruction is the recommended option [108,109]. Williams et al. showed that a simultaneous combined ACL reconstruction with a osteotomy to correct the varus alignment had superior short-term outcomes and a low complications rate [110]. Bonin et al. found that simultaneous combined ACL reconstruction and closed or open wedge HTO yielded satisfactory long-term outcomes [111].

During this fellowship, we had seen two cases of combined ACL reconstruction with high-tibia osteotomy. The key to the combined surgery is ensuring that the osteotomy plate does not block the position of the tibial tunnel during ACL reconstruction.

However, Latterman and co-authors have warned of the potential higher complication rates seen in such combined procedures. They had suggested that the procedure should be staged with the osteotomy performed followed by the ACL reconstruction in 9 to 12 months [112].

### Revision ACL

In the MARS group descriptive epidemiology of ACL revisions, the combination (37%) of a traumatic reinjury as well as technical reasons was the most common cause for the revision surgery [113]. Unlike the MARS group where more than 50% of revision ACL reconstructions were performed with allograft tissue, we saw the use of autograft tissue in revision ACL surgery– 2 BTB autograft and 1 hamstring autograft were used. We noted that in all cases that required revision, the femoral tunnel from the primary failed ACL reconstruction was placed too vertical and not in the described anatomical position. This meant that the revised femoral tunnel tunnel had to be drilled in a separate location and made the revision surgery less technically demanding.

**Figure 4:**
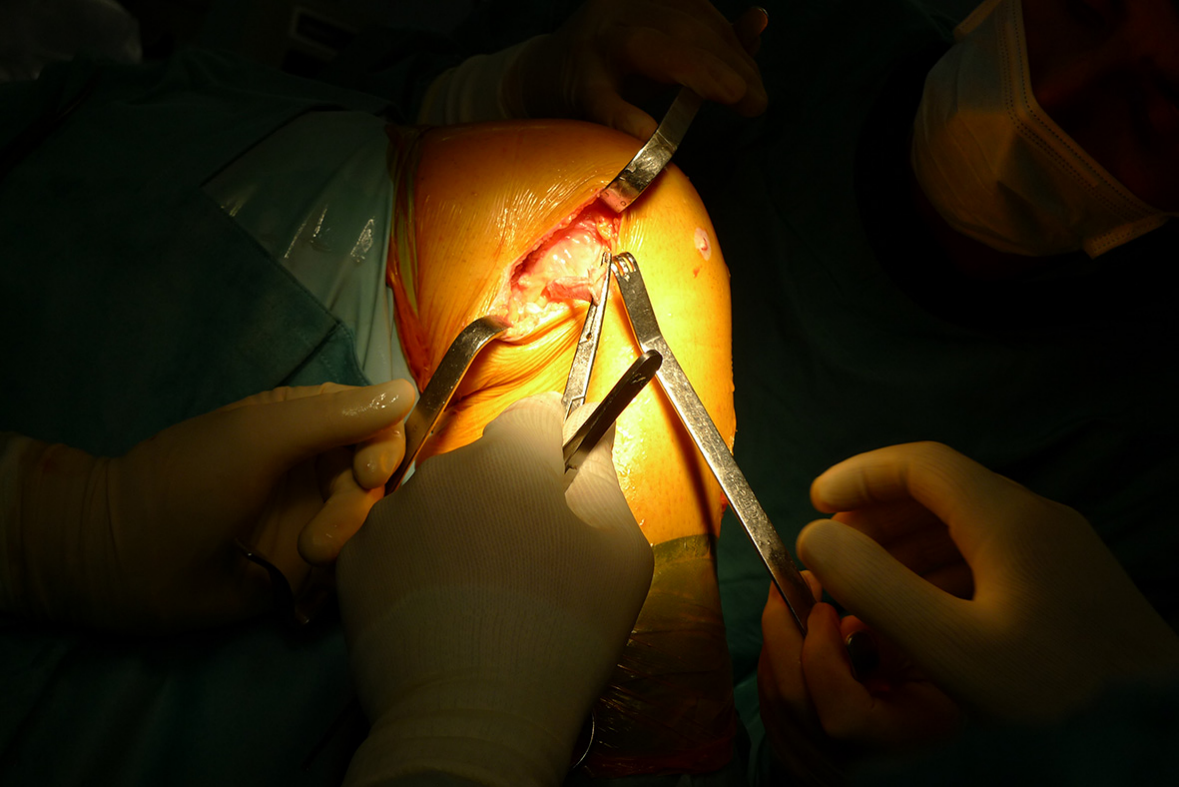
Extraarticular augmentation of iliotibial band during revision reconstruction of ACL.

In one centre, we also saw the use of an extraarticular augmentation of the iliotibial band (ITB) for revision anterior cruciate ligament.(Figure 4) This procedure start with the harvest of 1 cm wide and 20 cm long strip of iliotibial band, keeping the tibial attachment intact. The deep insertion of the ITB of the femur that lies posterior and proximal to the lateral epicondyle is identified. The ITB graft is fixed at this point in 30 degrees flexion and neutral internal rotation with a soft tissue washer screw. The graft is then looped back to the Gerdy tubercle and stitched down [114]. This technique is different from the extraarticular procedures in the 1980s where ITB grafts were routed beneath the fibular collateral ligament and sutured at the Gerdys tubercle [115,116]. These were non-anatomic placement of ITB grafts which do not restore the femoral tibial ITB attachments that resist internal rotation.

Two clinical studies by Noyes et al. and Ferretti et al. have shown that this extraarticular procedure added in ACL revision surgery significantly improves knee stability [114,116]. They believe that this extraarticular tenodesis procedure provides an additional restraint for tibial internal rotation and anterior translation.

## Conclusion

ACL Reconstruction techniques has evolved with our improved understanding of anatomy, kinematics, physiometrics, and surgical outcomes. The travelling fellowship provides us with the opportunity to learn these techniques and best practices from the various European sports centres and see if we can apply it to improve our own patient outcomes.

## Competing interests

The authors declare that they have no competing interests.

## Authors’ contributions

All authors (LYHD, RK, ZJZ, CKM) contributed, read and approved the final manuscript.
